# Novel serum proteomic biomarkers for early diagnosis and aggressive grade identification of prostate cancer

**DOI:** 10.3389/fonc.2022.1004015

**Published:** 2022-10-06

**Authors:** Ce Wang, Guangming Liu, Yehua Liu, Zhanpo Yang, Weiwei Xin, Meng Wang, Yang Li, Lan Yang, Hong Mu, Chunlei Zhou

**Affiliations:** ^1^ Department of Clinical Laboratory, Tianjin First Central Hospital, School of Medicine, Nankai University, Tianjin, China; ^2^ Department of Urology Surgery, Tianjin First Central Hospital, School of Medicine, Nankai University, Tianjin, China; ^3^ Department of Pathology, Tianjin First Central Hospital, School of Medicine, Nankai University, Tianjin, China

**Keywords:** data-independent acquisition, mass spectrometry, biomarker, aggressiveness, prostate cancer

## Abstract

**Background:**

Prostate cancer (PCa) is one of the most common tumors and the second leading cause of cancer-related death in men. The discovery of novel biomarkers for PCa diagnosis in the early stage, as well as discriminating aggressive PCa from non-aggressive PCa continue to pose a challenge. The aim of this study was to identify serum proteins that were sensitive and specific enough to detect early-stage and aggressive PCa.

**Methods:**

The serum proteomic profiling of patients with PCa and benign prostatic hyperplasia (BPH) was comprehensively analyzed using data-independent acquisition mass spectrometry (DIA-MS), and the bioinformatics analysis was performed. The differentially expressed proteins (DEPs) of interest were further verified by enzyme-linked immunosorbent assay (ELISA) and immunoturbidimetry assay.

**Results:**

Statistically significant difference in abundance showed 56 DEPs between early-stage PCa and BPH and 47 DEPs between aggressive and non-aggressive PCa patients. In addition, the verification results showed that serum L-selectin concentration was significantly higher (*p*<0.05) in Gleason 6 PCa when compared with BPH, and the concentration of osteopontin (SPP1) and ceruloplasmin (CP) increased with higher Gleason score.

**Conclusions:**

DIA-MS has great potential in cancer-related biomarker screening. Our data demonstrated that adding SPP1 and CP to PSA improved the separation of Gleason 7 (4 + 3) or above from Gleason 7 (3 + 4) or below compared with PSA diagnosis alone. Serum SPP1 and CP could be effective biomarkers to differentiate aggressive PCa (especially Gleason 7 (4 + 3) or above) from non-aggressive disease.

## Introduction

Prostate cancer (PCa) is the most frequently diagnosed cancer in men worldwide, with an increasing incidence observed over the past decade (1, 2). Most PCa patient present with indolent and slowly progressing tumor, while those with high-grade PCa tends to have bone metastasis, which in turn leads to disastrous complications and high mortality rates (3, 4).

The Gleason scoring system is the international standard for PCa classification that standardizes the risk assessment of patients with local tumor lesions based on histology (5, 6). It characterizes PCa into a score between 2 and 10. Cancers with lower Gleason scores (<7) tend to be less aggressive, while cancers with higher Gleason scores (especially >7) tend to be more aggressive (7). Gleason score (GS) 7 is reported as either GS 3 + 4 or GS 4 + 3. According to Epstein’s new grade group, patients with GS 7 (4 + 3) PCa are more likely to have inconsistent tumors during radical prostatectomy (RP) and higher biochemical recurrence rate after RP compared to GS 7(3 + 4) PCa (8). Prostate-specific antigen (PSA) is the most commonly used tumor biomarker, which has been approved for PCa screening by Food and Drug Administration (FDA) (9, 10). However, PSA does not perform well in distinguishing aggressive (AG) from non-aggressive (NAG) PCa phenotypes and other benign conditions (11, 12). Consequently, extensive research is still required to screen non-invasive biomarkers with better diagnostic and prognostic efficiency for PCa.

As is well known, proteomics technology based on mass spectrometry has been greatly improved, and it has contributed to many breakthroughs in disease-related biomarker discovery over the last decades (13, 14). Researchers have recently proposed a label-independent quantitative technology based on data-independent acquisition (DIA). Compared with the traditional data-dependent acquisition (DDA), DIA technology can scan all peptide parent ions in the interval, and quantify them through the peak area of the secondary ion signal, thus truly achieving panoramic, high-throughput, and high precision. Moreover, DIA does not need to remove high abundance protein and pre-fractionation, which ensure the authenticity and parallelism of the experimental results to the greatest extent (15). In 2015, Ruedi et al. identified and quantified 342 proteins by detecting the plasma proteome of 232 twins using DIA technology, which is conducive to discovering and evaluating clinical biomarkers (16). Subsequently, DIA technology was also applied to studying biomarkers for tumor diagnoses, such as ovarian cancer, renal cell carcinoma, colorectal cancer, lung disease, and others (17–20). Recently, several urinary or tissue biomarkers of PCa have been studied using DIA mass spectrometry (DIA-MS) (21, 22).

In the present work, we performed a proteomic study to analyze serum as a source for clinical biomarkers. We comparatively studied patients with PCa and benign prostatic hyperplasia (BPH) using DIA-MS technology and explored the invasiveness in GS 7 patients with different pathological manifestations. The main goal of this study was to discover novel biomarkers in serum that are sufficiently sensitive and specific to detect PCa in its early stage and to separate the AG PCa from NAG PCa and other benign conditions.

## Materials and methods

### Patients’ enrollment and serum sample collection

Serum samples of patients with suspected PCa before diagnosis were collected from Tianjin First Central Hospital, after which patients were enrolled into different groups based on histological findings by biopsy or surgical procedure. All PCa patients were initially diagnosed without other tumors. Patients without determined histology or had undergone surgery and chemotherapy as well as those who with other serious diseases were excluded. We firstly collected 30 BPH and 30 PCa for MS analysis. In order to further test and verify the candidate biomarkers, we collected another 35 BPH and 51 PCa for the ELISA and immunoturbidimetry verification.

All serum samples were aliquoted and stored at -80 °C until proteomic analysis. Each serum sample underwent no more than three freeze/thaw cycles before the test.

The use of clinical samples was approved by Tianjin First Central Hospital Institutional Review Board. All participants provided written informed consent, and all study cases were annotated with available clinical information in a manner that protected patient identities.

### Sample digestion

Serum samples were centrifuged at 5000g for 10 min, after which they were thawed to remove debris before processing for protein digestion. Serum samples were denatured with protein lysate without sodium dodecyl sulfate (SDS), reduced with dithiothreitol (DTT) to 10 mM final concentration for 30 min at 37°C, alkylated with iodoacetamide (IAA) to 55 mM final concentration, and reacted in the dark room at room temperature for 30 min. C18 columns were used to enrich the sample. Extract purified protein samples were re-dissolved with 25 μ L 50mm NH_4_HCO_3_, a vortex that oscillated for 1min, and was then briefly centrifuged for 1min. Next, protein extraction quality control was measured by Bradford Protein Quantification Kit and SDS-PAGE. After that, 100μg of protein from each sample was diluted with 50mM NH_4_HCO_3_ by 4 times volumes and digested with 2.5μg of trypsin enzyme in the ratio of protein: enzyme = 40:1 for 4 hours at 37°C. Finally, enzymatic peptides were desalted using a Strata X column and vacuumed to dryness.

### High pH reversed-phase separation

An equal amount of peptides was extracted from all samples for mixing, and the mixture was diluted with mobile phase A (5% ACN pH 9.8) and injected. The LC-20AB HPLC system (Shimadzu, Japan) coupled with a high pH C18 column (Gemini, 5μm, 4.6 x 250mm) was used. The sample was subjected to the column and then eluted at a flow rate of 1mL/min by gradient: 5% mobile phase B (95% ACN, pH 9.8) for 10 minutes, 5% - 35% mobile phase B for 40 minutes, 35% - 95% mobile phase B for 1 minute, flow Phase B lasted 3 minutes, and 5% mobile phase B equilibrated for 10 minutes. The elution peak was monitored at a wavelength of 214nm and the component was collected every minute. Components were combined into 10 fractions, which were then freeze-dried.

### DDA and DIA analysis by nano-scale liquid chromatography-tandem mass spectrometry

The dried peptide samples were reconstituted with mobile phase A (2% ACN, 0.1% FA) and centrifuged at 20,000g for 10 minutes, after which the supernatant was taken for injection. Separation was carried out by the UltiMate 3000 UHPLC liquid chromatograph (Thermo Fisher Scientific, San Jose, CA). The sample was first enriched in the trap column and desalted, then it entered a tandem self-packed C18 column (150μm, 1.8 x 35mm) and was separated at a flow rate of 500nL/min by the following effective gradient: 0~5 minutes, 5% mobile phase B (98% ACN, 0.1% FA); 5~120 minutes, mobile phase B linearly increased from 5% to 25%; 120~160 minutes, mobile phase B rose from 25% to 35%; 160~170 minutes, mobile phase B rose from 35% to 80%; 170~175 minutes, 80% mobile phase B; 175~180 minutes, 5% mobile phase B. The nanoliter liquid phase separation end was directly connected to the mass spectrometer as the following settings.

**Figure 1:**
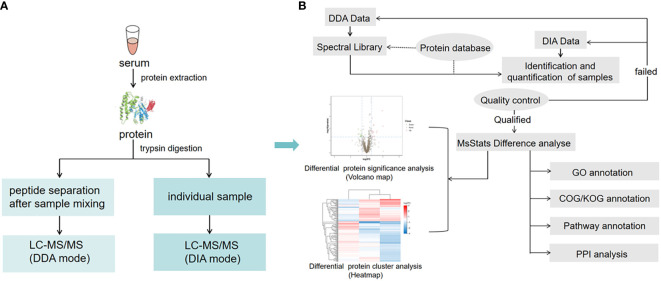
Serum sample DIA and DDA analysis workflow. **(A)** The main experimental workflow. Q-Exactive HF X was used to acquire mass spectrometry data for 60 samples in DIA mode. **(B)** Bioinformatics pipeline. This process is based on the sample data generated from the high-resolution mass spectrometer. Principal component analysis (PCA) and quantitative correlation of samples were also performed to evaluate the quality of the DIA data. For bioinformatics analysis, GO, COG, functional pathway annotation, and PPI analysis were performed in this study.

The workflow of DIA is shown in **Figure 1A**. Firstly, liquid chromatography (LC) separated peptides were ionized by nanoESI and injected into tandem mass spectrometer Q-Exactive HF X (Thermo Fisher Scientific, San Jose, CA) with DDA detection mode to construct a spectral library. Then, LC-separated peptides were ionized by nanoESI and injected in DIA detection mode. In this mode, the mass spectrometer was set to a wide precursor ion window to collect product ions in turn, thus, achieving complete collection of all detectable protein peak information in the sample and high-reproducible analysis of a large number of samples. Finally, identification and quantification of peptides and proteins were obtained from DDA spectral library by deconvolution of the DIA data.

### Bioinformatic analysis

Based on the sample quantitative results generated from a high-resolution mass spectrometer, the differential proteins between comparison groups were found. Finally, function enrichment analysis was performed. We also performed Gene ontology (GO), clusters of orthologous groups (COG), pathway functional annotation, and protein-protein interaction (PPI) analysis (**Figure 1B**).

### Validation of candidate biomarkers

The concentrations of candidate biomarkers in serum were measured. Serum PSA was measured by chemiluminescence (Roche Diagnostics, Germany), and ceruloplasmin was measured by immunoturbidimetry (Roche Diagnostics, Germany). L-selectin and osteopontin were validated using enzyme-linked immunosorbent assay (ELISA) kits purchased from Multisciences (Hangzhou, China) based on the double-antibody sandwich method according to the manufacturer’s instructions.

### Data analysis

For MS analysis, DDA data was identified by the Andromeda search engine within MaxQuant. mProphet algorithm was used to complete analytical quality control. MSstats software package based on linear mixed-effects models was used to perform differential analysis. A student t-test was used to estimate the significance of a change in the relative level (*p*<0.05). Kyoto Encyclopedia of Genes and Genomes (KEGG) pathway enrichment analysis was performed using phyper function in R software; Q value was obtained by Benjamini-Hochberg correction of a *p*-value.

For biomarkers verification, statistical analyses were done using SPSS Statistics version 24.0 (SPSS, Chicago, IL, USA) and GraphPad Prism 5 (GraphPad Software, La Jolla, CA, USA). Mann-Whitney tests were used to analyze the difference between two categories and stepwise associations between pathological findings of biopsy specimens (BPH or PCa with Gleason scores of 6, 7, or 8-9). The Kruskal–Wallis tests were used to analyze the differences among different GS groups. The predictive power of selected biomarkers was assessed using the receiver operating characteristics (ROC) curve. The value of the area under curve (AUC) was calculated as an indication of the accuracy of prediction. The ROC curves were generated and compared using GraphPad Prism 5. All *p* values were two-sided, with statistical significance set at *p*<0.05.

## Results

### Clinical characteristics of serum samples and proteomic profiles assessment

Clinical and pathological characteristics of serum samples from patients with histologically-proven PCa and BPH were analyzed. As shown in **Table 1**, 146 serum samples (60 for discovery and 86 for biomarkers validation) were recruited in this study. Included PCa patients represented all major histological subtypes at different pathological stages. The average age in the discovery cohort was 69.9 ± 7.7 years (range 56 to 85 years) and the average age in the validation cohort was 70.1 ± 7.5 years (range 44 to 86 years). There was no association between pathological stages and age.

**Table 1:** Clinical characteristics of serum samples in this study.

	Subtype		n (%)	Patients age (Mean ± SD)
Discovery cohort				
PCa (n=30)	GS 6		9 (30.0%)	69.0 ± 7.7
	GS 7	GS (3+4)	6 (20.0%)	69.2 ± 9.9
		GS (4+3)	5 (16.7%)	72.0 ± 7.4
	GS 8		4 (13.3%)	67.5 ± 4.5
	GS 9		6 (20.0%)	68.2 ± 6.7
BPH (n=30)			30	70.6 ± 8.3
Validation cohort				
PCa (n=51)	GS 6		13 (25.5%)	68.9 ± 6.8
	GS 7	GS (3+4)	16 (31.4%)	69.8 ± 6.5
		GS (4+3)	8 (15.7%)	63.0 ± 7.1
	GS 8		7 (13.7%)	66.4 ± 6.8
	GS 9		7 (13.7%)	68.7 ± 6.3
BPH (n=35)			35	71.2 ± 8.7

**Figure 2:**
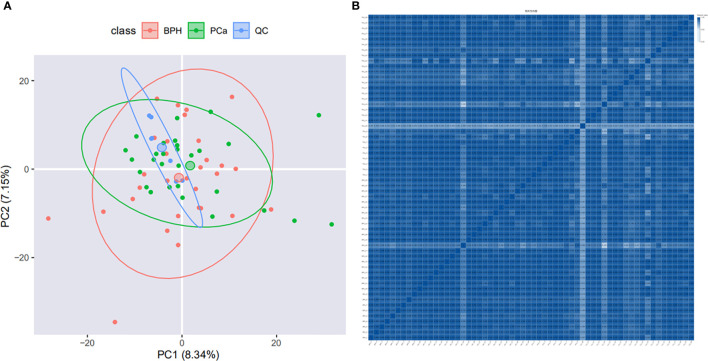
Quality control evaluation of DIA data. **(A)** PCA analysis. The X-axis is the first principal component and the Y-axis is the second principal component. **(B)** Heat map of sample correlation analysis. Both X and Y axes represent samples. The color represents the correlation coefficient; the deeper color represents the higher correlation.

PCA analysis showed that samples were relatively clustered, suggesting stable instrument performance and good data quality (**Figure 2A**). In order to determine the correlation of the protein quantification, the Pearson correlation of all protein abundances between every two samples was demonstrated by a heat map. The range of Pearson correlation was 0.192-1, on average. Pearson correlation of protein abundances between every two samples was > 0.5, which indicated a high correlation of protein expressed in all samples (**Figure 2B**). In this project, we quantified 11862 peptides and 1377 proteins under the DIA mode.

### Summary of the proteomic discovery of PCa and BPH

**Figure 3:**
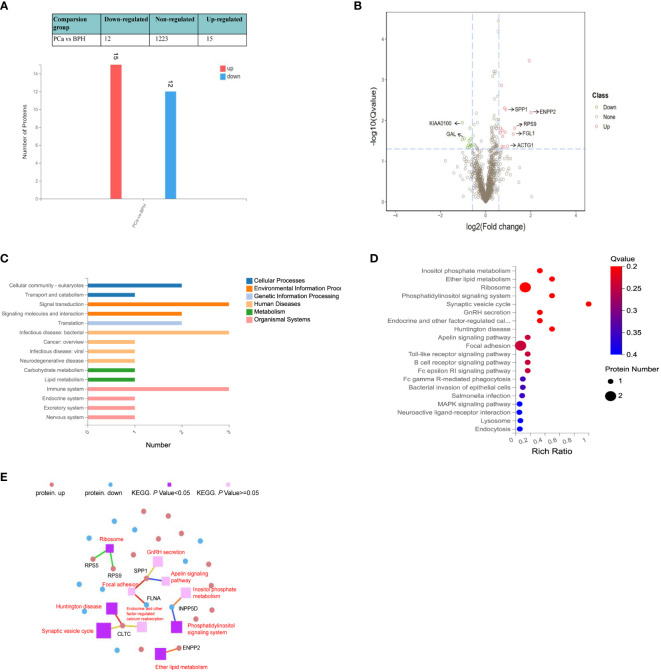
Quantitative differential analysis between PCa and BPH patients. **(A)** Bar chart of DEPs. The X-axis represents comparison group information; Y-axis represents the number of DEP. Red represents the significantly up-regulated proteins. Blue represents significantly down-regulated proteins. **(B)** Volcano map of the DEPs between PCa and BPH patients. Red and green dots represent up- and down-regulated proteins, respectively. **(C)** KEGG pathway classification. The X-axis represents the number of proteins annotated to a certain KEGG Pathway category, and the Y-axis represents the KEGG pathway category. **(D)** KEGG pathway enrichment analysis. The function of Q value < 0.05 was considered a significant enrichment. **(E)** KEGG pathway relationship network. The red and blue dots represent up-regulated and down-regulated differential proteins, respectively. The purple spheres represent the pathways of top 10 enrichment, dark color indicates significant enrichment, light color indicates insignificant enrichment, and the larger the area is, the higher the enrichment degree is.

We compared the serum proteome profiles between 30 PCa that contain different pathological stages and 30 BPH patients. MSstats software package was applied for each sample intra-system error correction and normalization. Based on the predefined comparison groups and the linear mixed effect model, the significance of differentially expressed proteins (DEPs) was subsequently evaluated. Three filtration criteria [fold change (FC) > 1.5, *p* < 0.05, and identified with at least 2 peptides] were used to obtain significant differential proteins. A total of 27 significantly differentially abundant proteins between PCa and BPH were identified, among which 15 proteins were up‐regulated and 12 down‐regulated (**Figure 3A**). Volcano map of significantly up-regulated five proteins include ectonucleotide pyrophosphatase/phosphodiesterase 2 (ENPP2), ribosomal protein S9 (RPS9), fibrinogen-like protein 1 (FGL1), osteopontin (SPP1), actin gamma 1 (ACTG1) and two down-regulated proteins potassium chan (KIAA0100), galanin (GAL) (**Figure 3B**).

Based on KEGG pathway analysis, we found the DEPs were mainly involved in signaling transduction, infection, and immune activity (**Figure 3C**). Different protein localizations were also found among DEPs. As shown in **Figure 3D**, the most enriched KEGG pathways were related to the ribosome (RPS5, RPS9), focal adhesion signaling pathways (FGL, SPP1), synaptic vesicle cycle (CLTC), and ether lipid metabolism (ENPP2), which were related to the invasion and metastasis of cancer. Metabolic pathways usually involve many proteins, and a single protein is often involved in multiple pathways. Thus, the KEGG Pathways with top 10 enrichment levels and their corresponding proteins were selected to demonstrate their relationship with the network diagram (**Figure 3E**). The results showed that both SPP1 and CLTC were involved in three pathways. SPP1 was involved in focal adhesion, GnRH secretion, and apelin signaling pathway, while CLTC was involved in calcium reabsorption and synaptic vesicle cycle.

### Identification of early PCa biomarkers using DIA-MS

**Figure 4:**
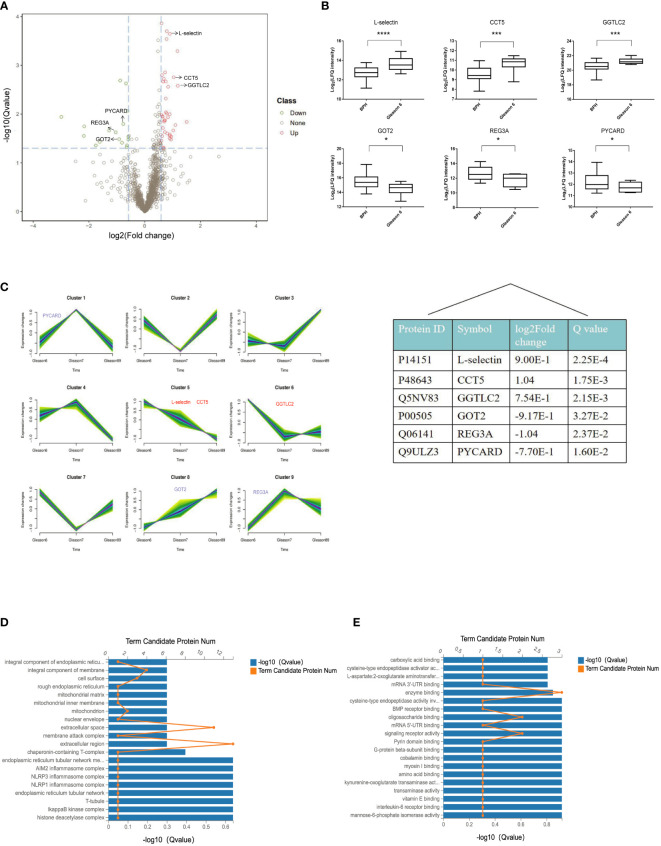
Quantitative differential analysis between early-stage PCa and BPH patients. **(A)** Volcano map of the DEPs between BPH and Gleason 6 patients. Red and green dots represent up- and down-regulated proteins, respectively. **(B)** Box plot analysis of intensity values for DEPs. (*p < 0.05, ***p < 0.005, ****p < 0.001). **(C)** The patterns of dynamic changes in DEPs during the progression of PCa analyzed by Fuzzy c-means clustering. The X-axis represents different Gleason scores of PCa, and the Y-axis represents log2-transformed intensity values in each cluster. **(D)** GO cellular component enrichment histogram. The column length of the X-axis below represents the size of the Q value[-log10(Q value)] and the value of points on the broken line on the top X represents the number of differential proteins annotated to this GO Term. **(E)** GO molecular function enrichment histogram.

To explore the value of DIA-MS in screening biomarkers of early-stage PCa, we compared the serum proteome profiles of early PCa patients (GS 6) with BPH in the discovery cohort. t-test analysis of label-free quantitation values (LFQ) intensity changes in the serum proteome between BPH and GS6 patients resulted in 56 proteins that were observed to be differentially abundant between GS 6 and BPH groups, with 39 significantly up-regulated and 17 down-regulated. A volcano map was made for significantly differential proteins with screening criteria of Q value< 0.05 and FC> 1.5, showing that L-selectin, T-complex protein 1 subunit epsilon (CCT5), and gamma-glutamyltransferase light chain 2 (GGTLC2) were significantly increased in Gleason 6 when compared with BPH, while glutamate oxaloacetate transaminase 2 (GOT2), pancreatitis-associated protein 1 (REG3A), and caspase recruitment domain-containing protein 5 (PYCARD) were significantly decreased in Gleason 6 (**Figure 4A**). Among these DEPs, median levels of L-selectin in GS 6 PCa patients were 1.9-fold higher than BPH (Q<0.05) (**Figure 4B**).

We further applied the Fuzzy c-means to cluster protein expression profiles in different Gleason scores PCa. In total, there were 9 distinct patterns representing differently regulated proteins. Among these, proteins in cluster 8 show an increasing trend with tumor progression; cluster 5 represent proteins that were down-regulated with tumor progression, whereas other clusters represent proteins displaying a bi-modal expression pattern. As for the DEPs between BPH and GS6 patients, our results showed that L-selectin and CCT5 fell into cluster 5, and GOT2 fell into cluster 8 (**Figure 4C**).

In order to gain better insight into the biological implication of the proteins with altered abundance, we analyzed the protein localization of DEPs. According to the cellular component, the most enriched were the extracellular region, extracellular space, and integral component of the membrane (L-selectin) (**Figure 4D**). As for molecular function, the three most enriched were enzyme binding, oligosaccharide binding, and signaling receptor activity, including L-selectin, GOT2, and REG3A (**Figure 4E**).

### DIA-MS identified candidate biomarkers of AG PCa and explored the aggressiveness of GS 7 PCa

**Figure 5:**
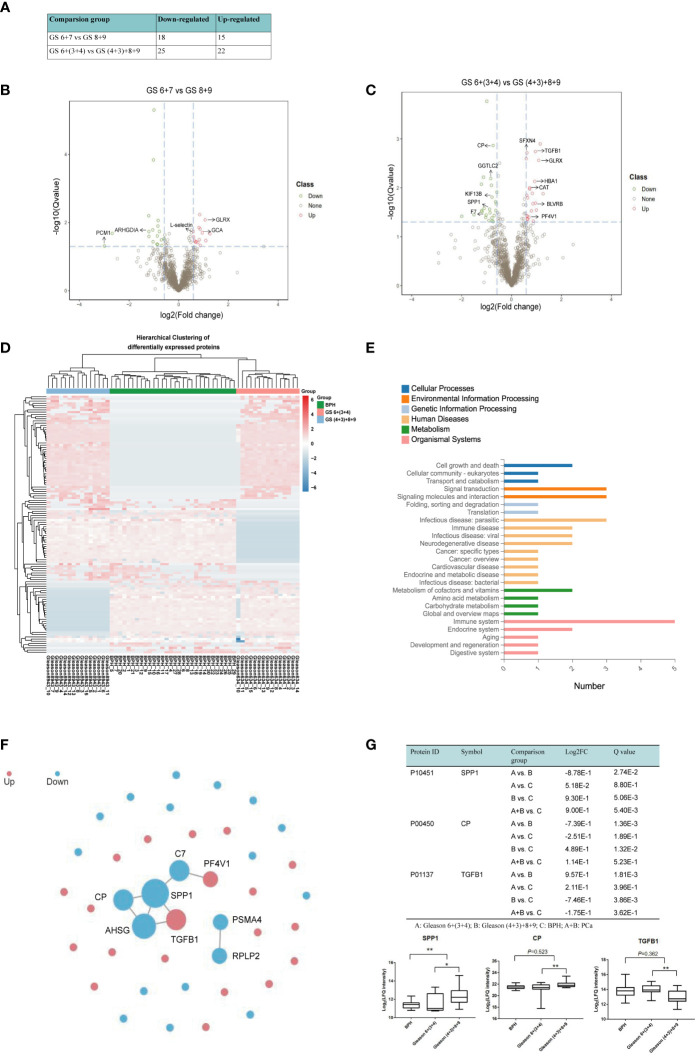
Quantitative differential analysis between AG and NAG patients. **(A)** Differential protein quantity of different comparison groups. FC >1.5 and Q value< 0.05 were selected by default for the significantly differential proteins. **(B)** Volcano map of the DEPs between GS 6 + 7 and GS 8 + 9 patients. Red and green dots represent up- and down-regulated proteins, respectively. **(C)** Volcano map of the DEPs between GS 6+(3 + 4) and GS (4 + 3)+8+9 patients. **(D)** The differences in DEPs were clustered. The X-axis represents the differential measure log2FC of the comparison group, and the Y-axis represents the protein. The redder the color block is, the greater the expression difference is, and the bluer the color is, the smaller the expression difference is. **(E)** The classification results of KEGG pathway annotation of differential proteins were plotted. The X-axis represents the number of proteins annotated to a certain KEGG Pathway category, and the Y-axis represents the KEGG Pathway category. **(F)** PPI network. Red represents up-regulated proteins, blue represents down-regulated proteins, and the circle size represents relationship intensity. **(G)** Box plot analysis of intensity values for selected proteins among BPH, GS 6+(3 + 4), and GS (4 + 3)+8+9. (*p < 0.05, **p < 0.01. A: GS 6+(3 + 4); B: GS (4 + 3)+8+9; C: BPH; A+B: PCa.

Currently, the Gleason score based on prostate biopsy is used to differentiate AG from NAG PCa. Cancers with GS <7 tend to be less aggressive, and cancers with GS >7 tend to be more aggressive; however, the aggressiveness of GS 7 in PCa patients has not yet been clearly determined. Due to the tumor heterogeneity, GS 7 PCa can be GS 3 + 4 or GS 4 + 3. When dividing GS 7 and GS 6 into one group and GS 8 and GS 9 into another, 33 significantly differentially abundant proteins were identified, of which 15 were up‐regulated and 18 down‐regulated. When we stratified GS 7 into GS 3 + 4 and GS 4 + 3 and then combined GS 7 (4 + 3) with GS 8 + 9 into the AG PCa group and GS 6 and 7 (3 + 4) into the NAG PCa group, 47 proteins were prioritized as DEPs between AG and NAG PCa patients, among which 22 proteins were up‐regulated and 25 down‐regulated (**Figure 5A**). The volcano map also showed more significantly DEPs when compared GS 6+(3 + 4) with GS (4 + 3)+8+9 (**Figures 5B, C**). Among these DEPs, 7 proteins, including platelet factor 4 variant (PF4V1), sideroflexin 4 (SFXN4), biliverdin reductase B (BLVRB), hemoglobin A1 (HBA1), catalase (CAT), glutathione peroxidase 1 (GPX1), and transforming growth factor beta-1 (TGFB1) were found to be up-regulated, while 5 proteins including GGTLC2, ceruloplasmin (CP), SPP1, kinesin-like protein (KIF13B), and coagulation factor (VIIF7) were down-regulated (**Figure 5C**). Hierarchical clustering was performed for 117 DEPs to verify if the AG PCa could be discriminated from NAG PCa patients and patients with BPH. As shown in **Figure 5D**, the DEPs performed good separation among these comparison groups. The KEGG metabolic pathways analysis between GS 6+(3 + 4) and GS (4 + 3)+8+9 indicated that the major significantly differentially abundant proteins involved the processes of immune activity (SPP1, TGFB1, PF4V1), signal transduction, and molecules and interaction (SPP1, TGFB1, CAT, PF4V1), infection (SPP1, TGFB1, HBA1), cell growth and death (CP, TGFB1) and metabolism of cofactors and vitamins (CP, BLVRB) (**Figure 5E**).

As proteins interact with each other to form complexes and perform their functions, we further analyzed the PPI of DEPs by comparing it with the STRING database. The interaction analysis of the differential proteins was carried out, and SPP1, CP, and TGFB1 were found at the center of the network module analysis (**Figure 5F**). Box plot analysis of SPP1, CP, and TGFB1 is shown in **Figure 4E**. We observed that SPP1 and CP were significantly higher in AG PCa while TGFB1 was lower in AG PCa compared with the NAG PCa group. SPP1 also remarkably increased in PCa samples when compared with the BPH group (**Figure 5G**).

### Verification of candidate biomarkers

**Figure 6:**
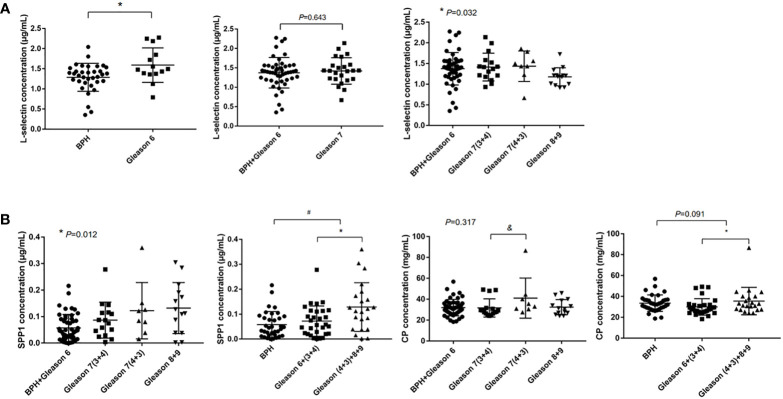
ELISA and immunoturbidimetry verification. The data were shown as mean ± SEM [*p < 0.05, #: BPH vs. PCa, &: GS 7(3 + 4) vs. GS 7(4 + 3)]. **(A)** The serum L-selectin concentration and **(B)** Serum SPP1 and CP concentration.

Based on protein classifier candidates identified in the discovery DIA experiment and bioinformatic analysis analysis, three candidate proteins (SPP1, L-selectin, and CP) were selected to detect early-stage PCa as well as differentiate AG PCa from NAG conditions. Serum from another 86 patients (51 for PCa with all types of PCa and 35 patients with BPH) was collected for further immunoturbidimetry and ELISA validation. As shown in **Figure 6A**, serum concentration of L-selectin was significantly higher in GS 6 PCa compared with BPH (1.592 vs. 1.288 µg/mL, *p*<0.05), which has similar trend to that identified by DIA-MS analysis. However, there was a dramatic decrease in PCa with higher Gleason score. And we did not find significant difference between BPH combined and GS 6 with GS 7.

In order to evaluate the difference in aggressiveness between GS (3 + 4) and GS (4 + 3), we compared SPP1 and CP among BPH combined with GS 6, GS 7(3 + 4), GS 7(4 + 3) and GS 8, 9 groups, significant differences were observed in serum SPP1 concentration among comparison groups (0.057 vs. 0.087 vs. 1.122 vs. 1.132 µg/mL, *p*=0.012), which indicated a increasing trend of the SPP1 concentration in PCa with higher Gleason grade. We also found significantly higher of CP concentration in GS 7(4 + 3) when compared with GS 7(3 + 4). We further stratified the GS 7 into GS (3 + 4) and GS (4 + 3), then combined GS 7 (4 + 3) with GS 8, 9 into an AG PCa group, and GS 6 and GS 7 (3 + 4) into a NAG PCa group. Our results showed higher concentration of SPP1 (0.129 vs. 0.072 µg/mL, *p*<0.05) and CP (35.582 vs. 29.960 mg/dL, *p*<0.05) in AG PCa versus NAG PCa (**Figure 6B**). Moreover, we also found higher serum SPP1 concentration in PCa patients compared with BPH (0.096 vs. 0.058 µg/mL, *p*<0.05).

In order to assess the potential clinical utility, we investigated whether the addition of selected biomarkers has the potential to provide additional information in the differentiation of PCa and AG PCa. The multivariate logistic regression analysis including age, PSA and SPP1 was made to compare PCa and BPH with age regarded as confounding factor. The final logistic model comprised of PSA and SPP1 could be expressed as follows:

Y=-0.147PSA-7.19SPP1-1.145

**Figure 7:**
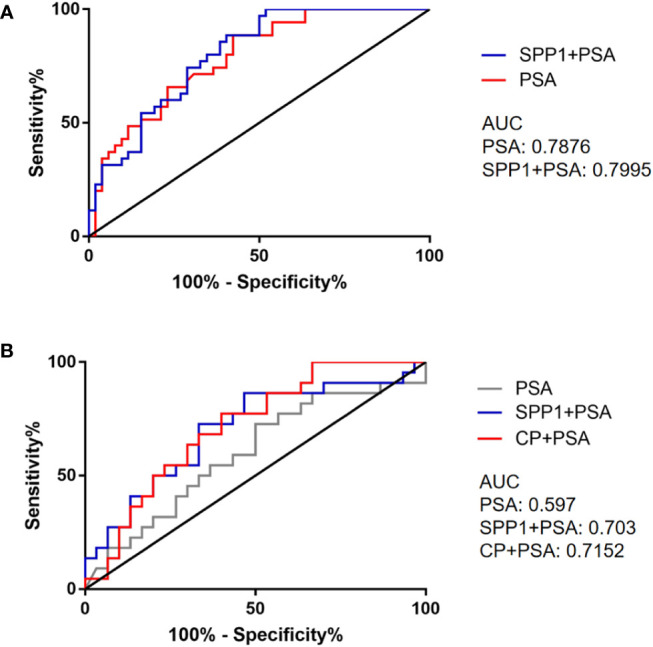
ROC curves of selected biomarkers. **(A)** ROC curves of PSA and combined detection of SPP1 in separating PCa and BPH. **(B)** ROC curves of PSA and combined detection of SPP1, CP in separating AG and NAG.

Then models of PSA and combined SPP1 with PSA were compared by ROC curves, the results showed that differences among the AUCs of PSA and combined PSA with SPP1 were not statistically significant (0.7876 vs. 0.7995) (**Figure 7A**). Then we made a similar analysis in the differentiation of Gleason score of 7 (4 + 3) and above with the Gleason score of 6 and 7 (3 + 4), the multivariate logistic regression analysis included age, PSA, SPP1, CP. The logistic models were constructed with age regarded as cofounding factor. We further combined SPP1, CP with PSA to differentiate the heterogeneity of pathological Gleason 7 (3 + 4) and (4 + 3) by ROC curves analysis. Among them, CP combined with PSA had the best performance (AUC=0.7152), followed by SPP1 combined with PSA (AUC=0.703), and PSA (AUC=0.597) (**Figure 7B**).

## Discussion

Most clinically diagnosed PCa represent indolent and non-aggressive tumors that grow slowly and are unlikely to cause significant symptoms, while in some cases, they may be aggressive with a greater risk of developing metastasis and other serious complications when definitive clinical intervention is more urgent compared with NAG tumor (23). Moreover, considering the relatively high mortality of AG conditions, it is critical to implement active surveillance (AS) at the early stage of PCa with the help of sensitive and objective diagnostic measurements. The Gleason scoring system, which has been considered a “gold standard” for grade PCa, is used to predict the behavior of PCa and evaluate its treatment outcomes. PCa with Gleason 2-6 is considered non-aggressive, while Gleason scores > 7 indicate aggressiveness with higher risk; the aggressiveness of GS 7 remains very controversial. In our previous comparative study of serum fucosylated PSA in PCa cases, we demonstrated that fucosylated PSA enriched by certain lectin could be an effective biomarker for differentiating AG [especially for GS≥7 (4 + 3)] from NAG PCa (24). Several studies have also shown that the increasing proportion of Gleason pattern 4 in the prostatectomy specimen has a close relationship with higher rates of biochemical recurrence and worse PCa-specific mortality and overall survival (25, 26), which indicated that GS 7 (4 + 3) significantly differs from GS 7 (3 + 4) in aggressiveness. Despite intense research, evaluation of the PCa aggressiveness and early-stage PCa diagnosis is still regarded as one of the most challenging problems for patients’ management and treatment because of the extremely heterogeneous morphology of PCa.

Human serum can be obtained by minimally invasive methods, which contain much biological information reflecting the physiological and pathological status of the body. Generally speaking, MS-based proteomics can obtain specific and quantitative information on all proteins without bias. However, the serum is a complex biochemical matrix with a wide and unbalanced dynamic range of proteins, which makes studying serum proteomics based on MS more challenging and might hinder the discovery of new candidate biomarkers. Thus, serum usually needs to be pretreated before MS analysis, which includes the removal of high abundance protein and pre-fractionation that could also lead to result bias (18). DIA-MS can overcome this problem as it can scan all peptide parent ions in the interval and quantify them by the peak area of the secondary ion signal. Removing high abundance protein is also unnecessary, thus ensuring the authenticity and parallelism of experimental results to the greatest extent. In addition, previous studies have reported that repetitive thawing and freezing could significantly change serum proteomes. In the present study, serum samples were subjected to no more than three freezing/thawing cycles before analysis in order to avoid errors and deviation in protein abundance analysis (27).

In the present study, we took advantage of high identification numbers, good repeatability, excellent quantitative accuracy, and a short cycle of DIA-MS to generate comprehensive proteomic profiling resource data on PCa with different Gleason scores and BPH samples. Our preliminary comparative study of serum from PCa and BPH patients revealed a panel of biomarkers mainly involved in signaling transduction, infection, and immune activity. Among these DEPs, L-selectin is a leukocyte adhesion molecule mainly expressed by lymphocytes and has a well-established association with thrombosis and inflammatory reactions (28). Still, few studies have reported the association between L-selectin and PCa. In this study, we observed a higher concentration of serum L-selectin in GS 6 PCa when compared with BPH in DIA-MS analysis. The ELISA validation experiment also showed that L-selectin concentration was significantly higher in the GS 6 PCa. Interestingly, we observed significantly decreased concentration in AG PCa samples. Although a previous study showed that increased expression of L-selectin ligand is correlated with the high metastatic potential of PCa cells (29), the expression of L-selectin in serum and other body fluids of PCa patients has rarely been reported and requires further clinical verification.

In order to further discriminate AG from NAG PCa and BPH patients, hierarchical clustering was performed by 118 DEPs. As expected, these DEPs performed an excellent separation among these comparison groups. According to the KEGG analysis, SPP1 was found to be related to several pathways, including focal adhesion and the apelin signaling pathway. SPP1 is a phosphorylated glycoprotein secreted by tumor cells and other host cells, which has been reported to be closely associated with the invasion, metastasis, and proliferation ability of certain cancers (30, 31). Some studies have shown that the dysregulated apelin, identified as a target gene of miR-224, may be associated with tumorigenesis and aggressive progression of PCa. Meanwhile, focal adhesion kinase was found to be increased in PCa patients with metastatic features (32, 33). We also regarded SPP1 as a differentially expressed protein between PCa and BPH by DIA-MS analysis in the present study. We further verified SPP1 in PCa and BPH samples by using the ELISA method, finding that SPP1 did not increase in the early stage of PCa, while significantly higher concentration was observed in the AG group when combining the GS 7 (3 + 4) group with GS 6 and the GS 7 (4 + 3) group with GS 8, 9, which was consistent with the MS analysis. And we also found significantly higher concentration in PCa compared with BPH. SPP1 has an important role in the formation and development of bone, early immune response, and bone remodeling processes (34). Most patients with AG PCa tend to develop bone metastasis. The increased activity of osteoclast and osteoblast exacerbate bone metabolism and bone loss, leading to the up-regulation of SPP1, which indicates that SPP1 might be a biomarker for AG PCa. CP was found at the center of the PPI network module analysis with SPP1. CP is an acute phase reactive protein that regulates the effect of antioxidants and oxidase activity and can catalyze the oxidation of polyphenols and polyamine substrates. The bioinformatics analysis showed that CP was involved in cell growth and death, which was closely related to cancer. CP has also been reported to be related to the malignancy and aggressiveness of several cancers (35, 36). Our validation results obtained by immunoturbidimetry also showed that CP serum concentrations were positively associated with the Gleason grade of PCa. A higher CP concentration was found in patients with GS 7 (4 + 3) or above. Multivariate logistic regression revealed that adding SPP1 and CP to PSA improve the separation of Gleason 7 (4 + 3) or above from Gleason 7 (3 + 4) or below compared with PSA diagnosis alone.

The main limitation of the present study was the sample size, which was limited, especially for GS 7 (3 + 4) and GS 7 (4 + 3). Consequently, future larger patient population study is needed to further validate the reported results of the diagnostic values of candidate biomarkers. Nevertheless, the potential biomarkers for distinguishing AG PCa identified in the present study could be helpful for better screening and diagnosis of PCa. These data also indicate that DIA-MS has great potential in tumor-related biomarker screening.

## Conclusion

In this study, we comprehensively analyzed the serum proteomic profiling of PCa and BPH patients using DIA-MS. Our data demonstrated that SPP1 and CP improved the separation of GS 7 (4 + 3) or above from GS 7 (3 + 4) or below. Serum SPP1 and CP could be effective biomarkers to differentiate aggressive PCa (especially Gleason 7 (4 + 3) or above) from non-aggressive disease. However, the size of clinical samples used in this study was relatively limited, and selected biomarkers will need to be validated by a large independent clinical cohort before their use in clinical practice.

## Data availability statement

The raw data supporting the conclusions of this article will be made available by the authors, without undue reservation.

## Ethics Statement

The studies involving human participants were reviewed and approved by Tianjin First Central Hospital Institutional Review Board. The patients/participants [legal guardian/next of kin] provided written informed consent to participate in this study.

## Author contributions

CZ and CW designed this research. CW, GL, and YHL conducted the statistical analyses and drafted the manuscript. ZY, WX, MW, YL, LY, and HM extracted the data and processed the figures and tables. All of the authors critically reviewed the manuscript. All authors contributed to the article and approved the submitted version.

## Funding

This work was supported by the 2020 Tianjin Health Science and Technology Project, Science and Technology Talent Cultivation Project (KJ20009) and the Tianjin Key Medical Discipline (Specialty) Construction Project.

## Conflict of interest

The authors declare that the research was conducted in the absence of any commercial or financial relationships that could be construed as a potential conflict of interest.

## Publisher’s note

All claims expressed in this article are solely those of the authors and do not necessarily represent those of their affiliated organizations, or those of the publisher, the editors and the reviewers. Any product that may be evaluated in this article, or claim that may be made by its manufacturer, is not guaranteed or endorsed by the publisher.
